# Assessing the influence of affective attitudes, demography and blood donor status on organ donor registration active decisions in opt-out systems

**DOI:** 10.1177/13591053231208531

**Published:** 2023-11-16

**Authors:** Lee Shepherd, Ronan E O’Carroll, Eamonn Ferguson

**Affiliations:** 1 Northumbria University, UK; 2 University of Stirling, UK; 3 University of Nottingham, UK; 4 Department of Public Health and Primary Care, National Institute for Health and Care Research Blood and Transplant Research Unit in Donor Health and Behaviour, University of Cambridge, UK

**Keywords:** affective attitudes, opt-out consent, organ donor registration, organ transplantation, perceived benefits

## Abstract

In contrast to opt-in systems, relatively little is known about what influences whether or not people register a decision about organ donation in opt-out systems. We address this gap in the literature. Participants (*N* = 756) living in a country with opt-out consent (Wales, UK) provided information on demographics and blood donor status. Participants indicated whether they had opted-in or opted-out (i.e. active decision), or not registered a decision under the assumption of deemed consent. Subsequently, their negative emotional beliefs (or affective attitudes) towards organ donation were measured. Opting-in was associated with being younger, having donated blood and holding superstitious beliefs about donation (jinx). Disgust (ick factor) deterred opting-in, and bodily integrity concerns increased opting-out. Positive affective attitudes increased opting-in and deterred opting-out. Actively opting-in increases the likelihood of organs being transplanted, thus, modifying affective attitudes and targeting blood donors should enhance the number of donors available under opt-out systems.

## Introduction

Given the lack of solid organs available for transplantation across the world (Lewis et al., 2021), some countries have tried to tackle this by moving from opt-in to opt-out consent for organ donation (e.g. England, Scotland, Wales, the Netherlands). In opt-in systems, people are required to act to indicate they consent to their organs being removed for transplantation. In these systems people can opt-in to organ donation by carrying an organ donor card or registering as an organ donor. In contrast, in opt-out systems, people are assumed to have consented to their organs being available for donation unless they have taken action to indicate they do not want their organs to be transplanted (e.g. de-registered and opted-out). There are different ways that opt-out consent can be implemented. Some opt-out systems allow people to opt-in and opt-out of organ donation (Rosenblum et al., 2012b). As such, people can make an active decision to register that they (a) want their organs to be transplanted when they die (i.e. opt-in) or (b) de-register to indicate that they do not want their organs to be used for transplantation when they die (i.e. opt-out). People can also passively accept the default and not register a decision with the assumption that their organs will be used for transplantation (deemed consent). Most opt-out systems apply a soft approach, meaning that in cases of deemed consent, transplantation can only occur if permission is received from family members or longstanding friends (Rosenblum et al., 2012a).^1^

There is mixed evidence surrounding the effectiveness of opt-out systems (for a discussion, see Shepherd et al., 2023). Despite adopting opt-out policies, this does not solve the problem, there are still a substantial number of people waiting for organs in France, Belgium, Italy and Spain (European Directorate for the Quality of Medicines and Healthcare, 2021). Given that a large number of countries use opt-out consent, and this number is growing, it is important to consider how to improve organ transplantation rates within opt-out systems. One strategy to achieve this in opt-out systems with an opt-in and opt-out register may be to improve the number of people who actively opt-in. Indeed, research from an opt-out system with an opt-in and opt-out register (Wales, UK) suggests that 84% of families agreed to a deceased loved one’s organs being transplanted when the deceased actively opted-in, but only 61% of families agreed when the deceased had not registered a decision and was thus assumed to support donation under deemed consent (Noyes et al., 2019). As such, encouraging people in opt-out systems to actively opt-in rather than rely on the default option of deemed consent may increase family consent rates and thus the availability of donor organs. Therefore, research that guides what factors will influence active opt-in decisions under an opt-out system are of vital importance. While we know that emotional beliefs about organ donation (or affective attitudes) influence decisions under opt-in systems (Morgan et al., 2008; O’Carroll et al., 2011; Shepherd and O’Carroll, 2014a), very little research has been conducted on decision-making under opt-out. Here we explore, for the first time, if affective attitudes similarly influence registration decisions under a well-established opt-out system.

### Affective attitudes towards organ donation

Affective attitudes have been repeatedly shown to influence organ donation decisions under opt-in systems (Ferguson et al., 2019). People may hold various negative and positive affective attitudes towards donation (Morgan et al., 2008). Negative affective attitudes include beliefs that the body needs to be kept whole (bodily integrity), doctors may let people die to obtain their organs (medical mistrust), organ donation is disgusting (ick factor) and becoming a donor may bring bad luck (jinx). Perceived benefits are positive affective attitudes and consist of the belief that organ donation benefits the donor (e.g. donors are lifesavers). Importantly, research under opt-in systems has shown that people who have registered as an organ donor are (a) less likely than people who have not registered as a donor to hold these negative affective attitudes and (b) more likely to believe in the perceived benefits of organ donation (Morgan et al., 2008; O’Carroll et al., 2011). Moreover, affective attitudes predict whether people subsequently register as an organ donor (O’Carroll et al., 2016; Shepherd and O’Carroll, 2014a). In addition, experimental research suggests that asking people to consider the affective attitudes reduces their willingness to donate (Doherty et al., 2017). Therefore, these studies suggest that under opt-in systems negative affective attitudes act as a barrier and perceived benefits acts as a facilitator to registering as an organ donor.

It is important to note that a variety of factors have been found to predict organ donor registration. For example, demographic variables such as age, gender, ethnicity and religion were found to predict opting-in to organ donation in a newly established English opt-out system (Coe et al., 2023). However, demographics and normative beliefs are generally more distant predictors of organ donation in comparison to people’s attitudes (Falomir-Pichastor et al., 2013). Indeed, affective attitudes were found to be more likely to differentiate donors and non-donors than factors such as age, gender, level of social deprivation, knowledge and normative beliefs (O’Carroll et al., 2011). Although this research was conducted in a country that used an opt-in system at the time of data collection, it demonstrates the importance of affective attitudes as well as demographic factors. Therefore, it is important to consider the influence of affective attitudes in opt-out organ donation systems.

As mentioned above, most research in this area has been conducted in opt-in systems. Although this research is useful for indicating some potential barriers to registration, people’s perceptions of organ donation are likely to vary based on whether opt-in or opt-out consent is used (Davidai et al., 2012; McKenzie et al., 2006). Therefore, it is important to also consider research in opt-out systems and not simply generalize from opt-in systems to opt-out systems. A few studies have been conducted in countries that were planning to move from an opt-in to an opt-out consent system (England and Scotland, UK). However, at the time of the research the countries were an opt-in system. Research from Scotland found that the negative affective attitudes were higher in people who planned to opt-out than people who planned to opt-in or use deemed consent (Miller et al., 2019b) and that people who planned to opt-out were likely to have concerns about bodily integrity and medical mistrust (Miller et al., 2020). Interestingly, an additional qualitative study indicated that people who planned to opt-in or use deemed consent, when Scotland introduced opt-out legislation, were likely to state that donating organs saved lives (Miller et al., 2019a). Similarly, research from England has found that the negative affective attitudes were higher and perceived benefits lower in people who planned to opt-out than people who planned to opt-in or use deemed consent (Clark et al., 2023).

Although this research is informative, it assessed people’s organ donor registration plans before the change to an opt-out system rather than their actual behaviour when living under an established opt-out policy. Given there is often a gap between people’s plans to undertake an action and their subsequent actions (i.e. the intention-behaviour gap, Sheeran, 2002), participants in this initial research may plan to opt-in or opt-out but may not undertake this action when the legislation is introduced. This may be due to the emotions that participants were feeling towards organ donation when completing the study making them less accurate at predicting how they will act later when they are not feeling the emotion (i.e. hot-to-cold empathy gap, Loewenstein, 2005), the lack of opportunity to undertake the behaviour (i.e. opting in or out) at the time when rating their intention or their motivation changing by the time that the opportunity to undertake the behaviour arrives (for a discussion, see Sheeran and Webb, 2016). Moreover, given that people prefer the status-quo (Samuelson and Zeckhauser, 1988), the effects may be due to the *potential change* from opt-in to opt-out consent. Therefore, despite this initial research being valuable, further research is needed to assess the influence of the affective attitudes on organ donor registration behaviour in people who are living under a well-established opt-out consent system.

### The present study

While positive and negative affective attitudes have been found to predict organ donor registration decisions under opt-in systems (Morgan et al., 2008; O’Carroll et al., 2011, 2016; Shepherd and O’Carroll, 2014a) and people’s anticipated future registration decisions under a future opt-out system (Clark et al., 2023; Miller et al., 2019a, 2019b, 2020), we have very little information on people’s registration decisions under the operation of an actual well-established opt-out system. It is not sufficient to generalize from these existing intention data and therefore, we assessed the role of positive and negative affective attitudes in predicting people’s registration decisions under a well-stablished opt-out system (Wales, UK).

Additionally, we know that blood donors are more likely to sign on the organ donor register under opt-in systems (Ferguson et al., 2018; Hyde et al., 2022). We explore if this generalizes to active opt-in decisions under an opt-out system. Similarly, given that age and sex have also been associated with organ donor registration in opt-in (O’Carroll et al., 2016) and opt-out systems (Coe et al., 2023), we also assessed the role of these demographic factors.

## Materials and methods

### Participants

In this manuscript, we analysed data that was collected as part of a study assessing next-of-kin approval of organ donation (Shepherd et al., 2023). However, in contrast to the original study, we assessed the factors that predicted whether people had registered as a donor (opted-in), registered as a non-donor (opted-out) or not registered a decision under the assumption of deemed consent (deemed consent). In the original study, an online survey provider (Qualtrics, https://www.qualtrics.com) was paid to recruit participants who were 18 years or older and currently living in Wales. For the current manuscript, we restricted the sample to only those participants who had been living in Wales when opt-out consent was first introduced (2015). This ensured all participants had been living in Wales for enough time to know it used opt-out consent. Participants completed the survey approximately 4 years after Wales introduced opt-out consent. As such, this was a well-established opt-out system. The sample consisted of 756 participants (*M*_age_ = 38.21, SD = 14.45; range = 18–76). There were 250 males (33.07%), 496 females (65.61%), four people who selected ‘Other’ (0.53%) and six people who preferred not to state their sex.

### Design

This element of the study used a correlational design. The predictor variables were the affective attitudes (bodily integrity, ick factor, medical mistrust, jinx and perceived benefits). The outcome variable was the participant’s organ donor registration status; specifically, whether they had opted-in, opted-out or used deemed consent. Age, sex and whether or not the participant had previously given blood were analysed as confounding variables.

### Materials and procedure

Ethical approval was obtained from the corresponding author’s Institutional Review Board. After giving consent, participants completed the measures below.

#### Health-based philanthropy

People can accurately self-report their blood (Bertalli et al., 2011) and organ donor registration status (O’Carroll et al., 2016). Therefore, we measured organ donor registration status by asking participants to indicate whether they had (i) registered a decision to donate their organs after they died (i.e. had opted in), (ii) registered a decision not to donate their organs after they died (i.e. had opted out) or (iii) not registered a decision about what should happen to their organs when they died (i.e. used deemed consent). Similarly, participants self-reported whether or not they had previously given blood (no; yes).

#### Affective attitudes towards organ donation

These were measured using a well-established scale (Morgan et al., 2008; O’Carroll et al., 2011). This scale contained two items measuring bodily integrity (e.g. ‘Removing organs from the body just isn’t right’; *r* = 0.81, *p* < 0.001), four items measuring medical mistrust (e.g. ‘Hospitals sometimes prescribe medication as a way of experimenting on people without their knowledge or consent’; *α* = 0.86), three items measuring the ick factor (e.g. ‘The thought of organ donation makes me uncomfortable’; *α* = 0.88), three items measuring jinx (e.g. ‘The surest way to bring about my own death is to make plans for it like signing an organ donor’; *α* = 0.77) and four items measuring perceived benefits (e.g. ‘Organ donors are heroic because they save lives’; *α* = 0.83). Each item was rated on a 7-point Likert scale (1 = *Strongly disagree*, 7 = *Strongly agree*). The mean of the items was used to calculate each of the five affective attitude subscales.

### Statistical analysis

The analyses were conducted in IBM SPSS (Version 28). First, correlation analyses were used to assess the associations between the predictor variables. Next, multinominal logistic regression analysis was used to assess the role of the affective attitudes (bodily integrity, medical mistrust, ick factor, jinx and perceived benefits) in predicting organ donor registration status (i.e. whether people had opted-in, opted-out or used deemed consent). Given the outcome variable had three categories, we needed to select a reference category. As mentioned above, family members are more likely to support donation decisions when the deceased has actively opted-in than when they have used deemed consent (Noyes et al., 2019). As such, encouraging people who are currently using deemed consent to opt-in could promote donation. Therefore, it was important to compare people who had opted-in with those using deemed consent in order to identify strategies to promote opting-in. Moreover, given that at the time of the study 41% of people in Wales had opted-in and 6% had opted-out (NHS Blood and Transplant, 2020), the vast majority of people were using deemed consent. Therefore, it was informative to compare the majority group of people using deemed consent with the two minority active decision groups (i.e. opting-in and opting-out). Because of this, deemed consent was the reference category (see Supplemental Online Material for reanalysis with alternative reference category). In this analysis, age, sex and blood donation status were also entered into the model as covariates. For the correlation and multinominal logistic regression analyses, participants who indicated ‘other’ or ‘prefer not to say’ when describing their sex were excluded to avoid the model being bias by categories containing low frequencies. One participant did not report their age and was also excluded from the analysis.

## Results

### Health-based philanthropy

As mentioned above, when the study was conducted data from the UK organ donor registry indicated that 41% of people in Wales had opted-in and 6% had opted-out (NHS Blood and Transplant, 2020). Importantly, these organ donor registration rates were similar to those within this study (see Shepherd et al., 2023). For example, we found that 56 participants (7.41%) had opted-out of organ donation, 317 (41.93%) had not registered a decision (i.e. deemed consent) and 383 (50.66%) had opted-in. This suggests that whether or not people took part in the study was unlikely to be influenced by whether they had opted-in, opted-out or used deemed consent. In addition, we found that there were 457 people (60.45%) who had not and 299 people (39.55%) who had previously given blood.

### Association between predictor variables

There were strong positive correlations between the negative affective attitudes (i.e. bodily integrity, medical mistrust, ick and jinx; Table 1). As expected, each of the negative affective attitudes was negatively associated with perceived benefits. Age was negatively associated with the negative affective attitudes, but positively associated with perceived benefits. Sex (coded 0 = male; 1 = female) was negatively associated with the negative affective attitudes, but positively associated with perceived benefits (for *t*-tests, see Supplemental Online Material). This indicates that males were more likely than females to hold these negative affective attitudes, but less likely to believe in the perceived benefits of organ donation. There was also a negative correlation between bodily integrity and blood donation (coded 0 = not previously donated blood; 1 = had previously donated blood). This indicates that people with bodily integrity concerns were less likely to be blood donors. Although there were some close associations between the predictor variables, further analysis revealed a lowest tolerance value of 0.33. Given that this value was above 0.20, including these predictors into a regression model was unlikely to create a multicollinearity issue (Menard, 1995).

**Table 1.** Descriptive statistics and intercorrelations between predictor variables.

	Descriptive statistics	1	2	3	4	5	6	7	8
(1) Age	*M* = 38.21	—							
	SD = 14.45								
(2) Sex	Female *n* = 496 (66.49%)	−0.08*	—						
	Male *n* = 250 (33.51%)								
(3) Blood donor status	Not donated *n* = 457 (60.45%)	0.12***	−0.06	—					
	Donated *n* = 299 (39.55%)								
(4) Bodily integrity	*M* = 2.88	−0.09*	−0.16***	−0.12**	—				
	SD = 1.79								
(5) Medical mistrust	*M* *=* 3.08	−0.15***	−0.11**	−0.04	0.57***	—			
	SD = 1.55								
(6) Ick factor	*M* *=* 2.87	−0.14***	−0.14***	−0.05	0.72***	0.68***	—		
	SD = 1.70								
(7) Jinx	*M* *=* 2.73	−0.22***	−0.17***	−0.01	0.59***	0.68***	0.70***	—	
	SD = 1.54								
(8) Perceived benefits	*M* *=* 5.36	0.16***	0.18***	0.04	−0.42***	−0.21***	−0.37***	−0.28***	—
	SD = 1.37								

The coding for the sex variable was male = 0 and female = 1. The coding for the blood donor status was not donated blood = 0 and donated blood = 1. For continuous variables, the descriptive statistics refers to the variable’s mean (*M*) and standard deviation (SD). For categorical variables, the descriptive statistics refer to the number and percentage of people within each category (percentages for gender do not include missing data).

* *p* < 0.05. **p < 0.01. ***p < 0.001.

### Affective attitudes predicting organ donor registration status

The multinominal logistic regression model explained a significant proportion of variance in organ donor registration status (Nagelkerke pseudo *R*^2^ = 0.37, χ^2^(16) = 273.38, *p* < 0.001).

#### Comparison of deemed consent with opting-out

People were more likely to have opted-out (compared to used deemed consent) if they (a) were older or (b) had bodily integrity concerns (Table 2). In contrast, people were less likely to have opted-out (compared to used deemed consent) when they believed the perceived benefits of organ donation. Therefore, opting-out is promoted by older age or holding bodily integrity concerns, but deterred by beliefs in the perceived benefits of organ donation.

**Table 2.** Multinominal regression analyses (*n* = 745).

	Deemed consent vs opt-out		Deemed consent vs opt-in	
	*B* (SE)	Odds ratio (95% CI)	*B* (SE)	Odds ratio (95% CI)
Age	0.03** (0.01)	1.03 (1.01, 1.06)	−0.02** (0.01)	0.98 (0.97, 0.99)
Sex	0.26 (0.35)	1.29 (0.66, 2.55)	−0.06 (0.19)	0.94 (0.65, 1.36)
Blood donor status	−0.76 (0.42)	0.47 (0.21, 1.06)	1.18*** (0.18)	3.27 (2.29, 4.66)
Bodily integrity	0.51*** (0.14)	1.66 (1.27, 2.16)	−0.34*** (0.08)	0.72 (0.61, 0.83)
Medical mistrust	0.08 (0.15)	1.08 (0.81, 1.44)	0.06 (0.09)	1.06 (0.90, 1.26)
Ick factor	0.07 (0.16)	1.08 (0.79, 1.47)	−0.24** (0.09)	0.79 (0.66, 0.94)
Jinx	0.14 (0.15)	1.15 (0.85, 1.55)	0.23* (0.09)	1.26 (1.05, 1.50)
Perceived benefits	−0.44** (0.15)	0.64 (0.48, 0.86)	0.33*** (0.07)	1.39 (1.20, 1.59)

The coding for the sex variable was male = 0 and female = 1. The coding for the blood donor status was not donated blood = 0 and donated blood = 1. 95% CI = 95% confidence intervals.

* *p* < 0.05. **p < 0.01. ***p < 0.001.

#### Comparison of deemed consent with opting-in

People were more likely to have opted-in (compared to used deemed consent) if they (a) were younger, (b) had previously donated blood, (c) held superstitious beliefs about organ donation (jinx) or (d) had beliefs in the perceived benefits of organ donation (Table 2). In contrast, people were less likely to have opted-in (compared to used deemed consent) if they (a) had bodily integrity concerns or (b) felt disgust towards organ donation (ick factor). Therefore, opting-in is promoted by being younger, having previously donated blood, holding superstitious views (jinx) and having beliefs in the perceived benefits of organ donation, but deterred by bodily integrity concerns and the ick factor.

## Discussion

This is the first study to explore the role of affective attitudes and people’s organ donor registration decisions under a well-established opt-out system. While previous research has shown that affective attitudes predict registration decisions under opt-in systems (Morgan et al., 2008; O’Carroll et al., 2011) and peoples’ potential registration plans when opt-out consent is introduced (Clark et al., 2023; Miller et al., 2019a, 2019b, 2020), we show for the first time that affective attitudes also influence organ donor registrations under a well-established opt-out system. We show that bodily integrity concerns motivated people to opt-out and deterred them from opting-in. Similarly, the ick factor deterred people from opting-in. By contrast, perceived benefits deterred people from opting-out and motivated them to opt-in. Therefore, these results provide strong evidence that under an opt-out system affective attitudes predict whether people opt-in, opt-out or use deemed consent.

Importantly, we also found that people who had opted-in or used deemed consent were not homogeneous, despite both supporting organ donation. Instead, people who opted-in and used deemed consent differed based on bodily integrity, the ick factor and perceived benefits. These results suggest that tackling bodily integrity concerns and the ick factor and promoting the perceived benefits of organ donation are vital for encouraging people to opt-in to organ donation in opt-out systems. This is important as families are far more likely to support relatives or friends’ decisions when they have actively opted-in (Noyes et al., 2019).

It is also important to consider the role of demographic variables. In contrast to previous research (Coe et al., 2023), sex did not predict whether people opted-in or opted-out. This may be due to attitudes being a more proximal predictor of organ donation behaviour than this demographic factor (Falomir-Pichastor et al., 2013; O’Carroll et al., 2011). However, we also found that older participants were more likely to have opted-out and were less likely to have opted-in, suggesting it may be more effective for organ donor registration campaigns to target younger people.

We also advance this area of research in other ways. For example, we extend research showing that in opt-in systems having previously donated blood increases the likelihood of registering as an organ donor (Ferguson et al., 2018) by demonstrating that people who have previously given blood are also more likely to opt-in under opt-out consent policy. Asking people who have previously donated blood to opt-in may, therefore, improve registration rates and subsequently relative consent rates under an opt-out systems. Perhaps surprisingly, we found that when controlling for the other affective attitudes, having superstitious beliefs towards organ donation (jinx) *increased* the likelihood of people opting-in. Interestingly, this effect only occurred after controlling for the other variables (i.e. the other affective attitudes). Similar positive associations have been found when assessing the relationship between jinx and families approving the transplantation of a deceased loved one’s organs (Shepherd and O’Carroll, 2014b), suggesting this effect is reliable. This may reflect the fact that people may hold both positive and negative attitudes towards an entity (Elliott et al., 2015). For example, superstitious beliefs may both oppose (e.g. ‘planning my death is bad luck’) and support registration decisions (e.g. ‘opting-in brings good karma’). After controlling for the other negative affective attitudes, jinx may reflect the superstitious beliefs that support organ donation. Therefore, people may opt-in to bring good karma, thereby making them feel some control over uncontrollable superstitions outcomes. As such, future research should measure both positive and negative superstitious beliefs in order to understand how these influence organ donation decisions.

### Strengths and limitations

Although these findings are important, they need to be considered alongside the strengths and weaknesses of this study. For the first time, we assessed the association between the affective attitudes and organ donor registration status (opted-in, opted-out and using deemed consent) in a large sample of participants from a country with a well-established opt-out system. Moreover, we used a well-validated measure of the affective attitudes (Morgan et al., 2008; O’Carroll et al., 2011). We also found the affective attitudes predicted organ donor registration status after controlling for age, sex and blood donor status. However, there were other demographic variables that we did not include in the study, such as ethnicity and religious beliefs. This is especially important given (a) that these factors have been found to be associated with people opting-in to organ donation (Coe et al., 2023) and (b) the over-representation of some ethic minority communities on the opt-out register (NHS Blood and Transplant, 2021). Therefore, it is important for future research to assess the role of affective attitudes in predicting whether or not people opt-out of organ donation in different ethnic groups and religious groups.

In addition, given the correlational design, we were unable to establish whether the affective attitudes predicted organ donor registration (i.e. whether people opted-in, opted-out or used deemed consent) or vice-versa. However, previous research has suggested that the affective attitudes predict subsequent organ donor registration behaviour (O’Carroll et al., 2016; Shepherd and O’Carroll, 2014a). Similarly, experimental research indicates a potential causal effect of the affective attitudes on organ donor behaviour via intention (Doherty et al., 2017). Therefore, it is likely that the affective attitudes predict organ donor registration. However, given that these previous studies were conducted in opt-in systems, further longitudinal and experimental research is needed in opt-out systems.

### Implications

Despite the limitations, this research has important implications. Opt-out systems have been introduced in numerous countries to improve organ transplantation rates and others are likely to follow. As mentioned above, previous research has found mixed results, with some studies indicating a beneficial effect of opt-out consent (e.g. Johnson and Goldstein, 2003) and other studies showing either no effect (Arshad et al., 2019) or mixed findings (Shepherd et al., 2014). In addition, opt-out countries still have a significant shortage of organs for transplantation (European Directorate for the Quality of Medicines and Healthcare, 2021). Therefore, in line with previous research (Noyes et al., 2019), we suggest that applying an opt-out system itself is insufficient to tackle the shortage of donor organs. Instead, other interventions need to be applied to promote organ donation. Importantly, this study demonstrates that under opt-out systems perceived benefit positively predicts opting-in, whilst bodily integrity and ick negatively predict opting-in. This suggests that interventions that influence these affective attitudes are needed to promote organ donor registration in opt-out systems. Such interventions should aim to promote the perceived benefits of donation, whilst tackling bodily integrity and ick concerns.

A recent review of behaviour change techniques for organ donor registration has suggested that interventions are likely to be effective when they include (a) information that tackles myths about donation, (b) information about the benefits to recipients and (c) instructions about how to register (Crawshaw et al., 2022). Interventions that include all three of these elements may be most effective in improving people’s affective attitudes. In opt-out systems actively opting-in rather than relying on the default option of deemed consent reduces the ambiguity about the deceased’s wishes (Shaw, 2017) and increases the likelihood that family members will allow the deceased’s organs to be transplanted (Noyes et al., 2019). By improving the affective attitudes, these interventions may increase the likelihood of people actively opting-in. This may subsequently increase family consent rates and thus availability of donor organs.

## Conclusion

In conclusion, under opt-out systems affective attitudes play a critical role in predicting whether people opt-in, opt-out or use deemed consent. Bodily integrity concerns were associated with people being more likely to opt-out and less likely to opt-in. Similarly, the ick factor was associated with people being less likely to opt-in. However, perceived benefits were associated with people being more likely to opt-in and less likely to opt-out. Implementing interventions that improve people’s affective attitudes towards organ donation is, therefore, likely to increase the number of people who actively register as a donor in opt-out systems. This has the potential to increase the availability of organs in opt-out systems and thus save lives.

## Supplemental Material

sj-doc-1-hpq-10.1177_13591053231208531 – Supplemental material for Assessing the influence of affective attitudes, demography and blood donor status on organ donor registration active decisions in opt-out systems Supplemental material, sj-doc-1-hpq-10.1177_13591053231208531 for Assessing the influence of affective attitudes, demography and blood donor status on organ donor registration active decisions in opt-out systems by Lee Shepherd, Ronan E O’Carroll and Eamonn Ferguson in Journal of Health Psychology

## Notes

1. In the UK, when an individual dies the organ donor register is checked to determine whether they had registered a decision to donate (opted-in) or not donate (opted-out). This decision is then discussed with family members to check that it was the deceased’s last known decision.
