# Supplementary material for: Assessing the influence of affective attitudes, demography and blood donor status on organ donor registration active decisions in opt-out systems
Source: J Health Psychol. 2023 Nov 16;29(8):825–35. doi: 10.1177/13591053231208531 (PMC11264565; doi:10.1177/13591053231208531)
Supplement: sj-doc-1-hpq-10.1177_13591053231208531 – Supplemental material for Assessing the influence of affective attitudes, demography and blood donor status on organ donor registration active decisions in opt-out systems [file sj-doc-1-hpq-10.1177_13591053231208531.doc]

**Supplemental Online Material**

**Effect of Sex and Blood Donor Status on Affective Attitudes**

As mentioned in the manuscript, sex and blood donor status were associated with the affective attitudes. Men were significantly more likely than women to believe each of the negative affective attitudes (Table S1). In contrast, women were significantly more likely than men to believe the perceived benefits of organ donation. Moreover, we found that bodily integrity concerns were higher in people who had not previously donated blood compared to people who had previously donated blood (Table S2).

**Multinominal Logistic Regression**

As discussed in the manuscript, deemed consent was used as the reference category in the multinominal logistic regression analysis. It is important to note that the results were very similar when the data was reanalyzed with opting-in as the reference category. In line with the initial analysis, people who had opted-out (compared to those who had opted-in) were older, held bodily integrity concerns and had lower perceived benefit beliefs (Table S3). The only additional finding was that the effect of blood donor status changed from non-significant in the original analyses with deemed consent as the reference category (*p* = .069) to significant in the reanalysis with opting-in as the reference category (*p* < .001).

**Tables**

**Table S1.** Influence of sex on the affective attitudes.

|  | Male  *M* (*SD*) | Female  *M* (*SD*) | t-test |
| --- | --- | --- | --- |
| Bodily integrity | 3.29 (1.87) | 2.70 (1.71) | *t*(744) = 4.31, *p* < .001, *d* = 0.33 |
| Medical mistrust | 3.33 (1.58) | 2.96 (1.51) | *t*(744) = 3.13, *p =* .002, *d* = 0.24 |
| Ick factor | 3.23 (1.69) | 2.71 (1.68) | *t*(744) = 3.97, *p* < .001, *d* = 0.31 |
| Jinx | 3.11 (1.63) | 2.54 (1.46) | *t*(744) = 4.81, *p* < .001, *d* = 0.37 |
| Perceived benefits | 5.02 (1.37) | 5.55 (1.32) | *t*(744) = 5.11, *p* < .001, *d* = 0.40 |

**Table S2.** Influence of donor status on the affective attitudes.

|  | Not previous donated blood  *M* (*SD*) | Previously donated blood  *M* (*SD*) | t-test |
| --- | --- | --- | --- |
| Bodily integrity | 3.05 (1.79) | 2.63 (1.75) | *t*(754) = 3.17, *p =* .002, *d* = 0.24 |
| Medical mistrust | 3.13 (1.49) | 3.01 (1.63) | *t*(754) = 1.00, *p =* .319, *d* = 0.07 |
| Ick factor | 2.94 (1.66) | 2.78 (1.75) | *t*(754) = 1.30, *p =* .195, *d* = 0.10 |
| Jinx | 2.75 (1.45) | 2.70 (1.67) | *t*(754) = 0.37, *p =* .709, *d* = 0.03 |
| Perceived benefits | 5.32 (1.34) | 5.44 (1.41) | *t*(754) = 1.18, *p =* .238, *d* = 0.09 |

**Table S3.** Multinominal regression analyses (*n* = 745). The coding for the sex variable was male = 0 and female = 1. The coding for the blood donor status was not donated blood = 0 and donated blood = 1. 95% CI = 95% confidence intervals.

|  | Opt-in vs. Opt-out | |  | Opt-in vs. Deemed consent | |
| --- | --- | --- | --- | --- | --- |
|  | B  (SE) | Odds ratio  (95% CI) |  | B  (SE) | Odds ratio  (95% CI) |
|  |  |  |  |  |  |
| Age | 0.05***  (0.01) | 1.05  (1.03, 1.08) |  | 0.02**  (0.01) | 1.02  (1.01, 1.03) |
| Sex | 0.32  (0.37) | 1.38  (0.67, 2.84) |  | 0.06  (0.19) | 1.07  (0.74, 1.54) |
| Blood donor status | -1.94***  (0.43) | 0.14  (0.06, 0.33) |  | -1.18***  (0.18) | 0.31  (0.22, 0.44) |
| Bodily integrity | 0.84***  (0.15) | 2.32  (1.74, 3.10) |  | 0.34***  (0.08) | 1.40  (1.20, 1.63) |
| Medical mistrust | 0.02  (0.16) | 1.02  (0.74, 1.39) |  | -0.06  (0.09) | 0.94  (0.79, 1.11) |
| Ick factor | 0.31  (0.17) | 1.37  (0.98, 1.92) |  | 0.24**  (0.09) | 1.27  (1.07, 1.52) |
| Jinx | -0.09  (0.17) | 0.91  (0.66, 1.27) |  | -0.23*  (0.09) | 0.80  (0.67, 0.95) |
| Perceived benefits | -0.77***  (0.15) | 0.46  (0.34, 0.63) |  | -0.33***  (0.07) | 0.72  (0.63, 0.83) |

* = *p* < .05, ** = *p* < .01 and *** = *p* < .001
